# PERINATAL FEATURES OF CHILDREN WITH AUTISM SPECTRUM DISORDER

**DOI:** 10.1590/1984-0462/;2017;35;2;00003

**Published:** 2017

**Authors:** Gabriela Foresti Fezer, Marília Barbosa de Matos, Angélica Luciana Nau, Bianca Simone Zeigelboim, Jair Mendes Marques, Paulo Breno Noronha Liberalesso

**Affiliations:** a Hospital Pequeno Príncipe, Curitiba, PR, Brasil.; b Universidade Tuiuti do Paraná, Curitiba, PR, Brasil.

**Keywords:** autistic disorder, child, infant, premature, asphyxia neonatorum, birth weight

## Abstract

**Objective:**

To analyze perinatal features of children with autism spectrum disorder (ASD).

**Methods:**

Retrospective review of the medical records of 75 children with ASD, between January 2008 and January 2015. Inclusion criteria were diagnosis of ASD based on DSM-5 criteria, and the informed consent form signed by the person who is legally responsible. The exclusion criterion was missing on the medical record. The variables analyzed were maternal age, prematurity (gestational age under 37 weeks), low birth weight (<2,500 g), and perinatal asphyxia (5^th^ minute Apgar score <7). Data were analyzed using the difference between proportions test, being significant *p*<0.05.

**Results:**

Seventy-five patients were included. Maternal age ranged from 21.4 to 38.6 years (29.8±4.1 years). Premature birth occurred in 14 (18.7%) patients, perinatal asphyxia in 6 (8.0%), and low birth weight in 32 (42.6%) patients. The prevalence of prematurity, low birth weight, and perinatal asphyxia among the children in our study was higher than the general prevalence of these conditions among all live births in our country, region, and state, which are, respectively, 11.5, 2.3, and 8.5% in Brazil; 11.0, 2.2, and 8.5% in Southern Brazil; and 10.5, 2.0, and 8.4% in the state of Paraná.

**Conclusions:**

Our findings show a higher prevalence of prematurity, low birth weight, and perinatal asphyxia among children with ASD. Some limitations are the retrospective study design, and the small sample size. Large prospective studies are needed to clarify the possible association between perinatal complications and ASD.

## RESUMO

**Objetivo:**

Analisar características perinatais de crianças com transtorno do espectro autista (TEA).

**Métodos:**

Revisão retrospectiva dos prontuários medicos de 75 crianças com TEA, entre janeiro de 2008 e janeiro de 2015. Os critérios de inclusão foram o diagnóstico de TEA baseado no DSM-5 e o termo de consentimento assinado pelo responsável legal. O critério de exclusão foi ausência de todos os dados no prontuário médico. As variáveis analisadas foram: idade materna, prematuridade (idade gestacional menor que 37 semanas), baixo peso ao nascer (<2.500 g) e asfixia perinatal (Apgar menor que 7 no quinto minuto). Os dados foram avaliados por meio do teste de diferença entre as proporções (nível de significância de p<0,05).

**Resultados:**

Setenta e cinco pacientes foram incluídos no estudo. A idade materna variou de 21,4 a 38,6 anos (29,8±4,1 anos). O parto prematuro ocorreu em 14 (18,7%) pacientes, asfixia perinatal em 6 (8,0%) e baixo peso ao nascer em 32 (42,6%). As prevalências de prematuridade, asfixia perinatal e baixo peso ao nascer entre as crianças com TEA neste estudo foram maiores do que as prevalências gerais dessas condições entre todos os nascidos vivos em nosso país, região e estado, as quais são, respectivamente, 11,5, 2,3 e 8,5% no Brasil, 11,0; 2,2 e 8,5% na região Sul e 10,5, 2,0 e 8,4% no estado do Paraná.

**Conclusões:**

Nossos achados mostraram maior prevalência de prematuridade, baixo peso ao nascer e asfixia perinatal em crianças com TEA. Algumas limitações são o desenho retrospectivo do estudo e a amostra de pequeno tamanho. Grandes estudos prospectivos são necessários para esclarecer a possível associação entre intercorrências perinatais e TEA.

## INTRODUCTION

Autism spectrum disorder (ASD) is a heterogeneous neurodevelopmental disorder characterized by impairment in social communication and interaction, and by restricted and repetitive behavior, interests and activities.^1^ ^,^ ^2^ ASD prevalence is not completely known worldwide, but it clearly has increased over the last 50 years.^3^ ^,^ ^4^ ^,^ ^5^ It is currently estimated at 0.1 to 2% among different populations, and affects more male than female population.^6^ The reasons for this ongoing rise are still a matter of discussion, and may include greater concern about ASD among parents, and health and education professionals, as well as broader diagnostic criteria. At the same time, an increase in the incidence of possible etiologic risk factors, explained in part by the improvements in obstetric and neonatal management, may contribute to numerous cases.^5^

The precise mechanisms of ASD development are unknown. Genetic and environmental factors interact, and reveal a multifactorial disorder.^7^ ^,^ ^8^ Obstetrical and perinatal factors have been associated to ASD.^5^ Advanced maternal and paternal age, maternal bleeding, cesarean section delivery, birth weight, low Apgar scores, hypoxia, prematurity, and congenital malformations are the most studied variables.^7^ Those factors contribute to focal brain inflammation that possibly correlates to ASD pathophysiology.^9^ However, this association remains inconsistent.

The dramatically increased survival rates for preterm infants in the previous decade highlight concerns regarding the long-term neurologic effects of preterm birth. Premature birth has repeatedly been found to increase the risk of ASD.^10^ ^,^ ^11^ ^,^ ^12^However, it is unclear whether ASD is related to prematurity itself or to prematurity-related comorbidities, since newborns who later develop an autistic disorder have increased rates of perinatal complications.^13^ ^,^ ^14^ This hypothesis is supported by the association between perinatal asphyxia and the development of ASD, which has been suggested in many studies.^11^ ^,^ ^12^ ^,^ ^14^ ^,^ ^15^ ^,^ ^16^ Anoxia caused by birth asphyxia, would excessively activate the dopaminergic system, and dopaminergic hyperactivity has been found in some autistic children.^15^ ^,^ ^16^

Low birth weight is a condition that has a direct association with prematurity. However, there is evidence that being a low birth weight neonate is an independent risk factor for the development of ASD, especially if the infant was small for gestational age (SGA).^17^ Conditions leading to poor intrauterine growth, like placental insufficiency, may contribute directly to an increased risk for ASD, or indirectly, through other associated conditions, such as intracranial hemorrhage.^11^ ^,^ ^17^

Given the rise in ASD prevalence, significant related morbidities and its impact in life quality, it is fundamental to investigate risk factors and possible etiologies that may contribute to an early diagnosis and intervention. The goal of our research is to describe perinatal features of children with ASD, which has been previously associated with its etiology

## METHOD

We retrospectively reviewed the medical records of all the children with ASD admitted to one of the Neuropediatrics’ ambulatory care centers of Pequeno Principe Hospital, in Curitiba, Brazil, between January 2008 and January 2015, evaluating them for perinatal features. This was a convenience sampling. In order to be included in the study, the participants had to have a diagnosis of ASD based on DSM-5 clinical criteria, established by an expert clinical evaluation, as well as the informed consent form signed by the person who is legally responsible. The exclusion criterion was missing on the medical record. All the 75 children admitted to the ambulatory met the criteria. Sample size calculation was not performed.

Clinical variables included maternal age at the time of the infant’s birth, prematurity, defined as gestational age under 37 weeks at birth, low birth weight (<2,500 g), and perinatal asphyxia, defined as Apgar score lower than 7 at the 5th minute after birth. These data were precisely registered on the patients´ medical records on the occasion of the first visit, and it was collected from the child´s personal health record in the hospital of birth. Other variables, regarding pregnancy and peripartum period, such as gestational bleeding, gestational diabetes, maternal infections, cesarean section, and abnormal presentation were not available in the patient´s medical records, and therefore were not analyzed in this study. We also did not have information about paternal age and some neonatal comorbidities, such as intracranial bleeding, seizures, or time spent in neonatal intensive care unit. All patients underwent magnetic resonance imaging (MRI) of the brain.

We then compared the prevalence of prematurity, low birth weight, and perinatal asphyxia among the children with ASD in our study, with the prevalence among all the live births in the year 2013 in the State of Paraná, as well as in the southern region of Brazil, and in the whole country. Statistical analysis was performed using the difference between proportions test (5% significance level). The tests were conducted by statistical software (STATISTICA, version 12, StatSoft, Tulsa, USA).

The local Ethics Committee on Research Involving Human Subjects (number registration - 44905015.0.0000.0097) approved all aspects of this research.

## RESULTS

From January 2008 to January 2015, 75 patients with ASD were admitted to one of the Neuopediatrics’ ambulatory care centers of Pequeno Principe Hospital, in Curitiba, Brazil, and all of them were included in the study. Of them, 28 (37.3%) were female and 47 (62.7%) were male. The age at diagnosis ranged from 12 to 58 months (33.7±12.2 months). Maternal age at pregnancy ranged from 21.4 to 38.6 years (29.8±4.1 years).

Premature birth occurred in 14 (18.7%) patients: birth at 26 weeks (3 patients; 4.0%), at 28 weeks (2 patients; 2.7%), at 30 weeks (1 patient; 1.3%), at 32 weeks (3 patients; 4.0%), at 33 weeks (1 patient; 1.3%), at 34 weeks (3 patients; 4.0%), and at 35 weeks (1 patient; 1.3%). Perinatal asphyxia occurred in 6 patients (8.0%). The two conditions were associated in three patients. Low birth weight occurred in 32 (42.6%) patients. Fourteen patients who were low birth weight newborns were also premature. None of the patients presented congenital malformations.

According to SINASC data, the prevalence of prematurity among all live births in Brazil, in Southern Brazil, and in the State of Paraná in the year 2013 was 11.5, 11.0, and 10.5%, respectively.^18^ There was a statistically significant difference between the prevalence of prematurity in Southern Brazil and in the state of Paraná, and the prevalence of prematurity in children with ASD (18,6%) in our study (*p*=0.044 and 0.034, respectively). Comparing the prevalence of preterm births in Brazil with the prevalence among children with ASD in the study, the prevalence of preterm birth was also higher among children with ASD, but there was no significant difference (*p*=0.055).

The estimated prevalence of perinatal asphyxia in Brazil, in Southern Brazil, and in the State of Paraná in the year 2013 was 2.3, 2.2, and 2.0%, respectively.^18^ When we compared these data with the prevalence of perinatal asphyxia among children with ASD in our study, we can see that the prevalence is higher among the children with ASD, with statistically significant differences for each of the regions compared (*p*=0.034, 0.032, and 0.028, respectively).

In the year 2013, 8.5% of the neonates in Brazil were born with less than 2,500 g. The prevalence was exactly the same in Southern Brazil, and very close in the State of Paraná (8.4%). When we compared these numbers with the prevalence of low birth weight infants in our study, we find very significant differences (*p*=0.000) for each of the regions analyzed. We also did the same analyses with the low birth weight newborns in our study group who were not premature (18 patients), and obtained the same results (*p*=0.000).

All patients included in the study underwent MRI examination of the brain. In 19 (25.3%) patients, the MRI was reported to be abnormal. Seventeen of these patients were premature and/or had suffered perinatal asphyxia. The follow abnormalities were found: cerebral atrophy (5 patients; 6.7%), agenesis of the corpus callosum (1 patient; 1.3%), multicystic encephalomalacia (3 patients; 4.0%), periventricular leukomalacia (3 patients; 4.0%), agenesis of the corpus callosum and cerebral atrophy (3 patients; 4.0%), hydrocephalus and cerebral atrophy (1 patient; 1.3%), periventricular leukomalacia and cerebral atrophy (1 patient; 1.3%), periventricular leukomalacia and gliosis in the left frontal lobe (1 patient; 1.3%), and periventricular leukomalacia and hydrocephalus (1 patient; 1.3%).

## DISCUSSION

The exact cause of autism spectrum disorder is unknown, but it is thought to be associated with an interaction of genes and environmental factors.^7^ ^,^ ^9^ Our findings showed a higher prevalence of prematurity, low birth weight, and perinatal hypoxia in individuals diagnosed with ASD, compared with the general population. This is in agreement with what has been demonstrated in previous studies.^9^ ^,^ ^11^ ^,^ ^19^

Advanced maternal age is one topic of interest often studied as a risk factor for ASD, and it is related to both genetic and environmental risk factors.^20^ Increased rates of chromosomal abnormalities and genomic modifications have been associated with older maternal age.^10^ ^,^ ^21^ ^,^ ^22^ In addition, older mothers have a less favorable *in utero* environment, due to endocrine and hormonal factors, which can lead to more obstetrical complications.^11^ ^,^ ^12^ ^,^ ^13^ ^,^ ^14^ ^,^ ^15^ ^,^ ^16^ ^,^ ^17^ ^,^ ^18^ ^,^ ^19^ ^,^ ^20^Finally, some studies theorize that men and women with a genetic predisposition for ASD may be more likely to delay childbearing until later years, and that older parents might be more health conscious, and might seek medical advice for children with developmental delay sooner than younger parents.^10^ ^,^ ^20^ ^,^ ^21^

A recent meta-analysis supported an association between advanced maternal age and risk of autism. The relative risk of autism in mothers aged 35 years or older compared with mothers aged 25-29 years was 1.52.^22^ In our sample, the mean maternal age was 29.8±4.1 years. Therefore, we can assume that advanced maternal age was not associated with ASD in our study.

Many studies have examined the association between prematurity and the development of ASD.^10^ ^,^ ^21^ ^,^ ^22^ ^,^ ^23^ However, the etiology of this association remains unclear. Some researches focus on the role of intrauterine inflammation as a link between prematurity and ASD.^9^ ^,^ ^23^ It is already known, for example, that maternal bacterial infection during pregnancy is a major cause of premature labor. Maternal infection results in inflammatory response that leads to the onset of spontaneous labor. The same inflammatory response that triggers labor can interfere on the development of the central nervous system (CNS)and therefore possibly contribute to ASD.^23^ Maternal stress during pregnancy is also a cause of preterm labor. This can be explained by the release of corticotrophin releasing hormone (CHR) in the hypothalamus, which occurs in response to stress, as demonstrated in studies that found increased serum levels of CHR in mothers who delivered preterm babies.^24^ ^,^ ^25^ CRH activates mast cells, which release several pro-inflammatory cytokines. All this cascade of events in the immune system could disrupt the gut-blood-brain barriers, and permit neurotoxic molecules to enter the brain, resulting in brain inflammation, thus contributing to ASD pathogenesis.^9^

On the other hand, it is unclear whether ASD is related to prematurity itself or to prematurity-related comorbidities.^13^ ^,^ ^14^ Newborns who later develop an autistic disorder have increased rates of perinatal complications. It has been suggested that early environmental insults may predispose subjects to autistic disorders by affecting brain development.^9^ ^,^ ^13^ ^,^ ^23^ A study in Sweden, which analyzed the correlation between autism and prematurity, showed reduction in the risk of autism when adjustment was done for maternal factors, and birth characteristics (small for gestational age and congenital malformations). It also found no association between autism and prematurity when adjustment was done for perinatal morbidity (low Apgar scores at 5th minute, intracranial bleeding, cerebral edema, or seizures in the neonatal period).^13^ This indicates that the association between preterm birth, and autistic disorders may be mediated by prenatal and neonatal complications influencing early brain development.

In our study, the prevalence of prematurity among autistic children was higher than the general prevalence of prematurity in our country, region, and state (*p*=0.055, 0.044, and 0.034, respectively). However, we did not have information about perinatal comorbidities, so we could not do an adjusted analysis for these factors.

Another fact that supports the idea that early brain insult could predispose to ASD is the association between perinatal asphyxia and ASD, suggested in many studies.^11^ ^,^ ^12^ ^,^ ^14^ ^,^ ^15^ ^,^ ^16^A study that compared twins with ASD, demonstrated that markers of hypoxia, such as respiratory distress or having an oxygen requirement at birth, were all associated with increased risk of ASD among concordant and discordant twin pairs (if both or only one twin had ASD). In discordant twins, if only one twin experienced a risk factor associated with a marker of hypoxia, it was always the twin with an ASD.^19^ Anoxia caused by birth asphyxia would excessively activate the dopaminergic system, and dopaminergic hyperactivity has been found in some autistic children.^15^ ^,^ ^16^ Perinatal asphyxia could also help to explain the male preponderance for ASD, since male infants typically suffer more neurologic dysfunction due to cerebral hypoxia, in comparison to females.^16^ ^,^ ^19^ In our study, we found a higher prevalence of perinatal asphyxia in the children of our sample comparing with the prevalence in Brazil, Southern Brazil, and the State of Parana, with statistically significant values (*p*=0.034; 0.032 and 0.028, respectively).

The prevalence of low birth weight infants in our study was much higher than the general prevalence in our country, region, and state (*p*<0.001). This is in accord with what has been demonstrated in previous studies.^10^ ^,^ ^11^ ^,^ ^15^ ^,^ ^26^ Limperopoulos et al.^26^ found a prevalence of ASD of 26.0% in infants with a birth weight lower than 1,500 g, compared with 5.7% positive screen prevalence in normal children. Lampi et al.^10^ and Moore et al.^27^ also showed an association between low birth weight and ASD. Moreover, they correlated birth weight with gestational age, and found a positive correlation between SGA infants and risk of ASD. In the study of Lampi et al., low birth weight infants (<2,500 g) had a 60.0% increased odds of autism (OR=1.60, 95%CI 1.05-2.30, *p*=0.029). SGA infants had a 70.0% increased odds for autism compared with adequate for gestational age infants (OR=1.70, 95%CI 1.10-2.60, *p*=0.009).^10^ Conditions leading to poor intrauterine growth, like placental insufficiency, may contribute directly to an increased risk of ASD, or indirectly, through other associated conditions, such as intracranial hemorrhage.

Still regarding birth weight, when we analyzed only the group of low birth weight infants who were not premature (18 patients), we also obtained significative differences comparing to the general population (*p*=0.000). These findings suggest that low birth weight may possibly be associated with ASD, independent of prematurity. We did not have access to data for SGA prevalence in our country, and therefore we did not include this variable in our analysis.

The 17 patients with prematurity and/or perinatal asphyxia in our study showed abnormalities on MRI. The emerging literature of structural and functional neuroimaging in autism may reveal some underlying abnormalities of the central nervous system.^28^ Periventricular leukomalacia and cerebral atrophy, for example, are injuries that are characteristic of premature patients. Therefore, the presence of these findings in the MRI of patients with ASD strengthens the association between autism and prematurity.

Our findings suggest that prematurity, perinatal asphyxia, and low birth weight may be associated with ASD, strengthening the theory that early brain injury is involved in the pathogenesis of autism. This is of prime importance, since these conditions are modifiable risk factors. However, the fact that this is a retrospective study and we had a convenience sample, a small sample size, and did not have a control group should lead to some limitations in our conclusions. Also, confounding factors that we could not evaluate must be considered. In fact, one of the difficulties in studying perinatal features is that many of the variables are not independent from each other. Large prospective, population-based studies may help to confirm the findings of our study, as well as to identify modifiable risk factors, to then allow early interventions, aiming at the reduction of the current increasing prevalence of ASD.
